# Spinal Cord Ischemia After Lower Extremity Surgery in Pediatric Osteogenesis Imperfecta With Thoracic Kyphoscoliosis: Tertiary Care Center Experience in Jeddah, Saudi Arabia

**DOI:** 10.7759/cureus.31599

**Published:** 2022-11-17

**Authors:** Bandar M Hetaimish, Ahmed Q Alturkistany, Hassan A Ahmed, Eid A Almasoudi, Abubakr S Alzwaihri

**Affiliations:** 1 Department of Orthopedic Surgery, University of Jeddah, Jeddah, SAU; 2 Department of Orthopedic Surgery, King Faisal Specialist Hospital & Research Centre, Jeddah, SAU; 3 College of Medicine, University of Jeddah, Jeddah, SAU

**Keywords:** saudi arabia, pediatric, paralysis, kyphoscoliosis, osteogenesis imperfect

## Abstract

Osteogenesis imperfecta is a rare genetic disorder of type 1 collagen which primarily affects children and leads to recurrent bone fractures. In addition, spinal abnormalities can also occur. We report a case of a 13-year-old male with osteogenesis imperfecta type III, associated with severe femur deformity and thoracic kyphoscoliosis, who developed neurological injury after lower extremity surgery. The patient was in a supine position when general anesthesia was administered. The operation lasted for approximately 250 minutes, and anesthesia for 310 minutes, with an estimated blood loss of 600 cc. Apart from a low mean arterial pressure value (45 mm Hg) intraoperatively, the procedure was uneventful. Early postoperatively, he developed spinal paralysis at the level of T4-T7, and an MRI of the spine demonstrated high signal intensity within the spinal cord from level T3 to T7. Subsequently, he was admitted to the pediatric intensive care unit for further assessment and management. Follow-up revealed recovery of paralysis after 12 months.

## Introduction

Osteogenesis imperfecta (OI), also called brittle bone disease, is a rare collagen type 1 genetic condition. The affected genes, COL1A1 and COL1A2, are responsible for the disease's entire clinical symptoms ‎[1]. The illness is characterized by blue sclera with recurrent bone fractures; another crucial characteristic of the condition is spinal abnormalities, which can affect up to 80% of people with OI. Scoliosis is significantly connected with age, according to new data from the literature. According to Liu et al. [2], another distinctive factor promoting the development of scoliosis is joint hyperlaxity, which is observed in these patients. OI damages not only the bone but also the spine and vertebrae by altering collagen, increasing the likelihood of vertebral collapse and, as a result, progressive scoliosis [3-5]. In patients with OI, spine abnormalities such as scoliosis, kyphosis, and even kyphoscoliosis can develop as a result of trauma. To the best of our knowledge, this is the first case report of a patient with OI with kyphoscoliosis who developed spinal cord ischemia after surgery aimed to correct his lower limb deformity.

## Case presentation

**Figure 1:**
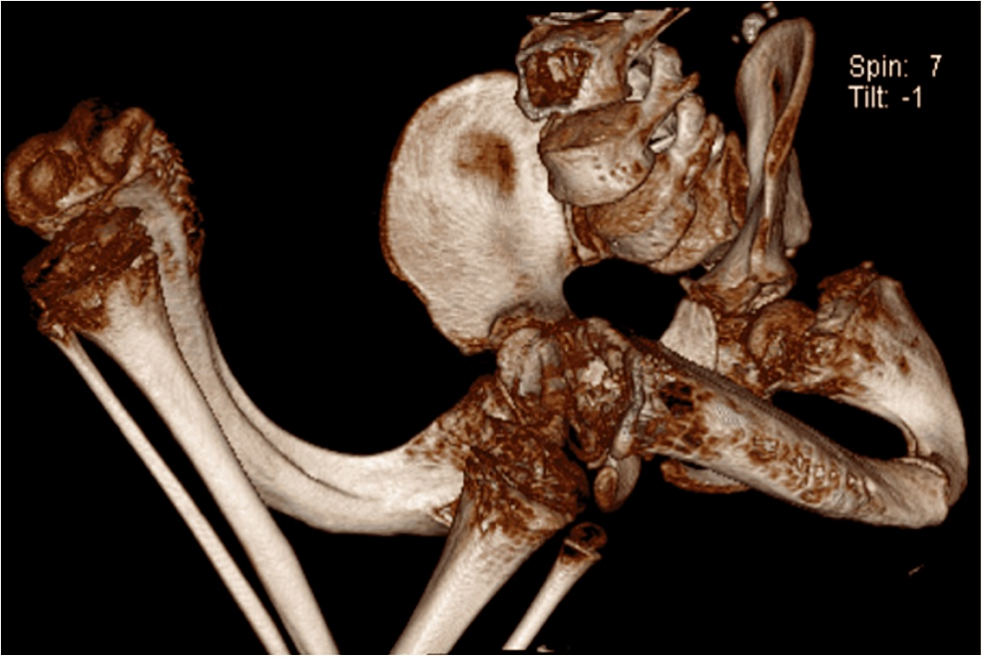
2D CT showed significant lower extremity deformity

This is a case of a 13-year-old boy with osteogenesis imperfecta type III associated with severe femur deformity and thoracic kyphoscoliosis. The patient has no family history of thrombotic disease. A preoperative neurologic examination was negative. Preoperative computed tomography (CT) showed significant lower extremities deformity (Figure 1). The apex of thoracic kyphosis was at T4. The type of operation was left femur corrective.

Under general anesthesia, the patient was in a supine position with padding of bony prominences on a radiolucent table. Preoperatively, the patient received cefazolin 760 mg intravenously. Fluoroscopic-guided two incisions were utilized, one proximal to the tip of the greater trochanter (GT) for guide wire insertion and the second incision at the osteotomy site through a direct lateral approach. The incision was made after making the osteotomy site through intraoperative fluoroscopic images. Femoral shaft osteotomy was at the point of maximum deformity. The guide wire was inserted from the tip of the GT until it reached the point of the osteotomy. The osteotomy was then performed, and the guide wire was advanced. Then, the bone started to fracture and split, and bleeding increased, followed by dropping in the patient's blood pressure. The decision was made to insert a temporary rush nail and bring the patient back for the second stage for Fassier Duval (FD) rod insertion.

The time for general anesthesia was administered to the patient in the supine position; 321 minutes were spent on anesthesia and 250 minutes on the procedure. Intraoperatively, neuromonitoring was not used. Intraoperatively, the lowest mean arterial pressure (MAP) values (45 mm Hg) were recorded. Oxygen (O_2_) saturation was kept at 100% during the treatment, resulting in an estimated blood loss of 600 cc. The urine output (1.2-1.7 mL/kg/hour) was within normal ranges. Operative time, anesthetic duration, intraoperative events, estimated blood loss, O_2_ saturation, mean arterial pressure (MAP), urine output, patient positioning, neurological deficit onset time, and neurological recovery time were all included in our outcome measures. 

**Figure 2:**
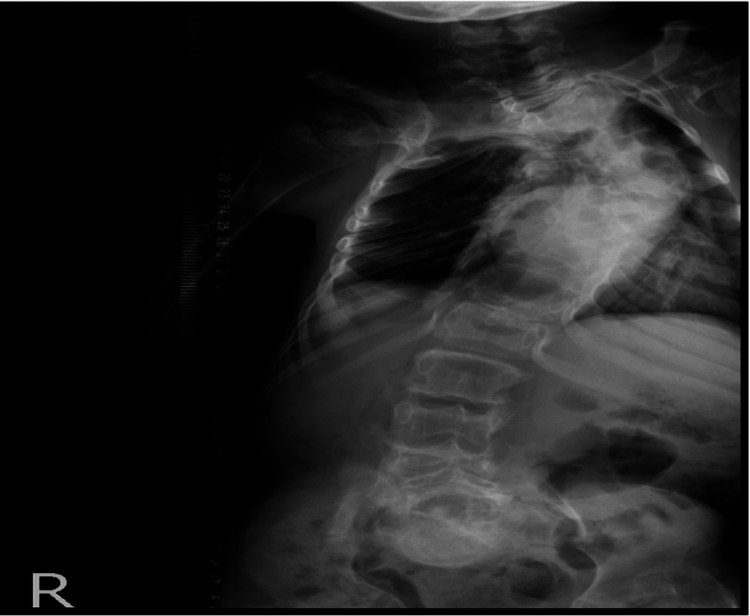
X-ray showed significant spinal kyphoscoliosis deformity

**Figure 3:**
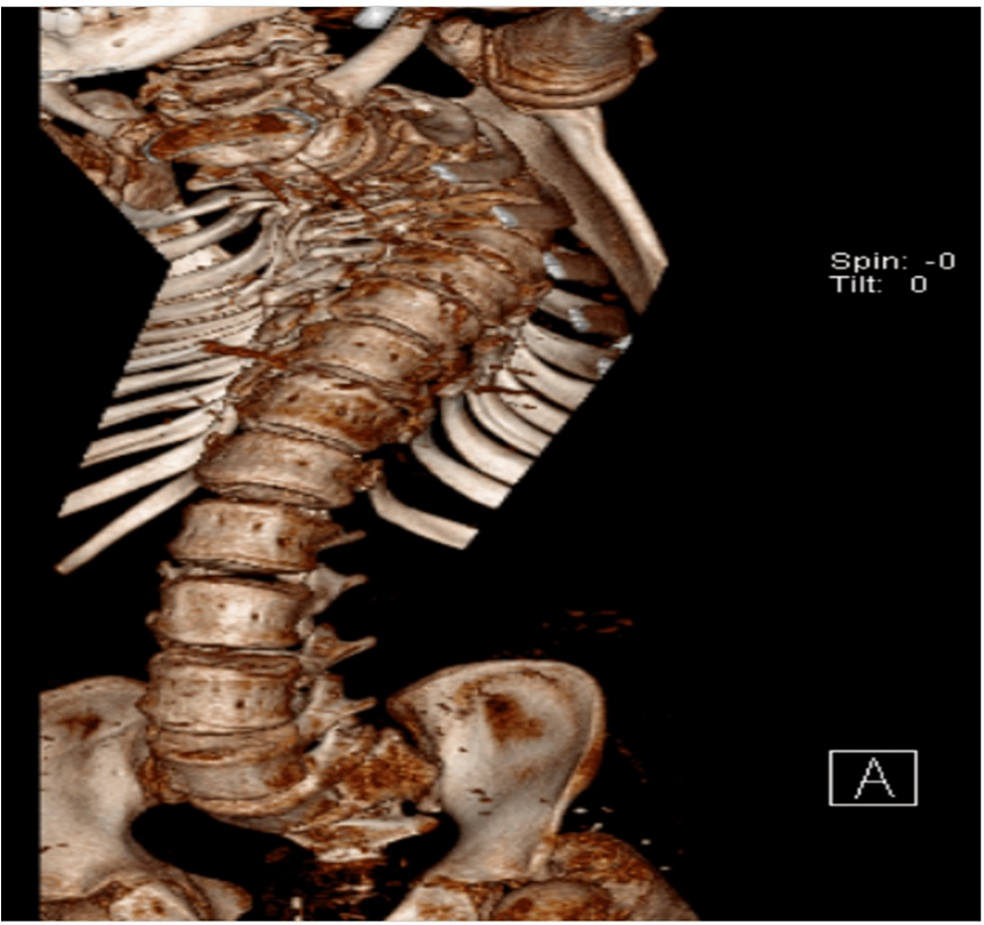
2D CT showed significant spinal kyphoscoliosis deformity

**Figure 4:**
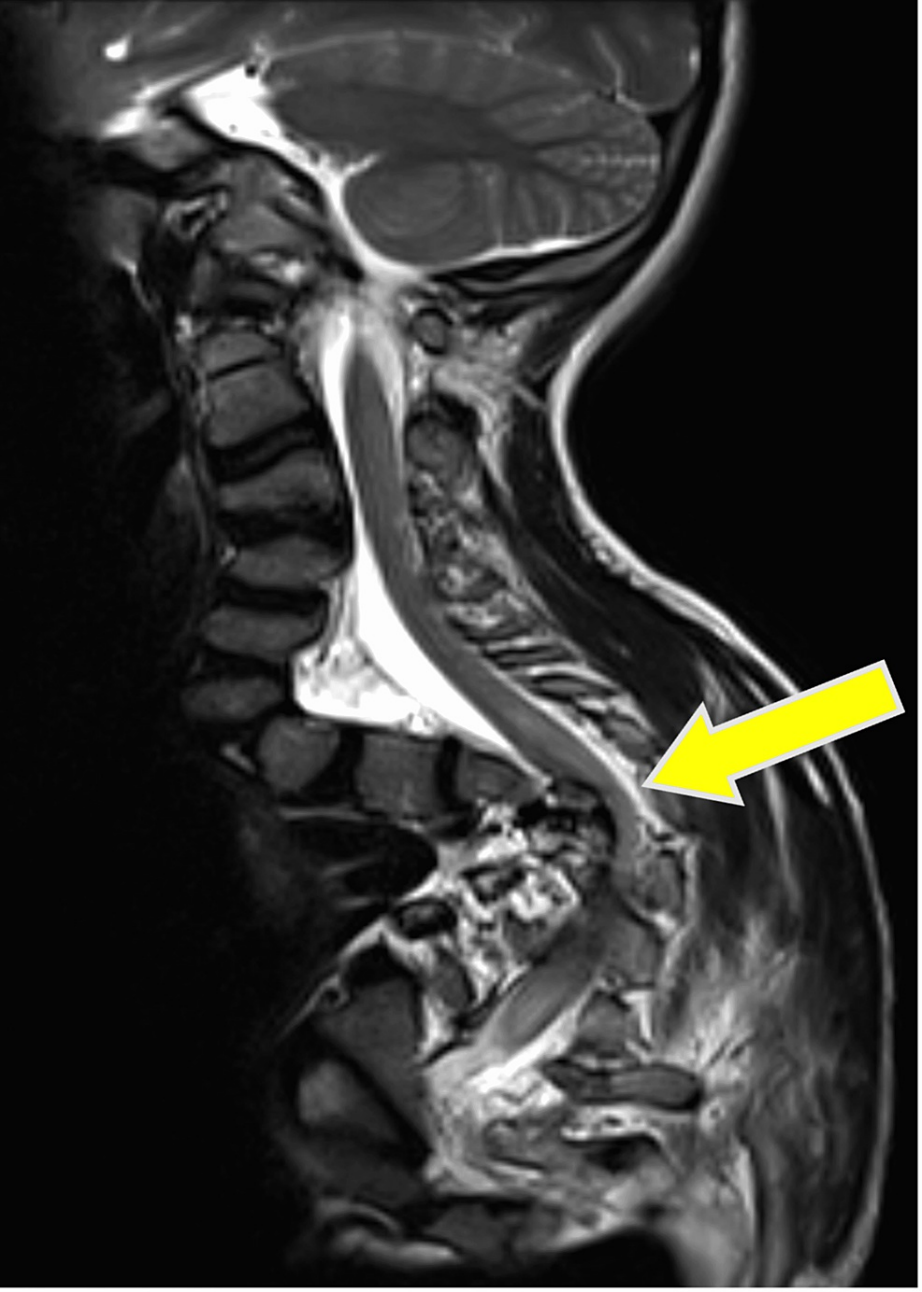
MRI showed high signal intensity within the spinal cord from the level T3 to T7

**Figure 5:**
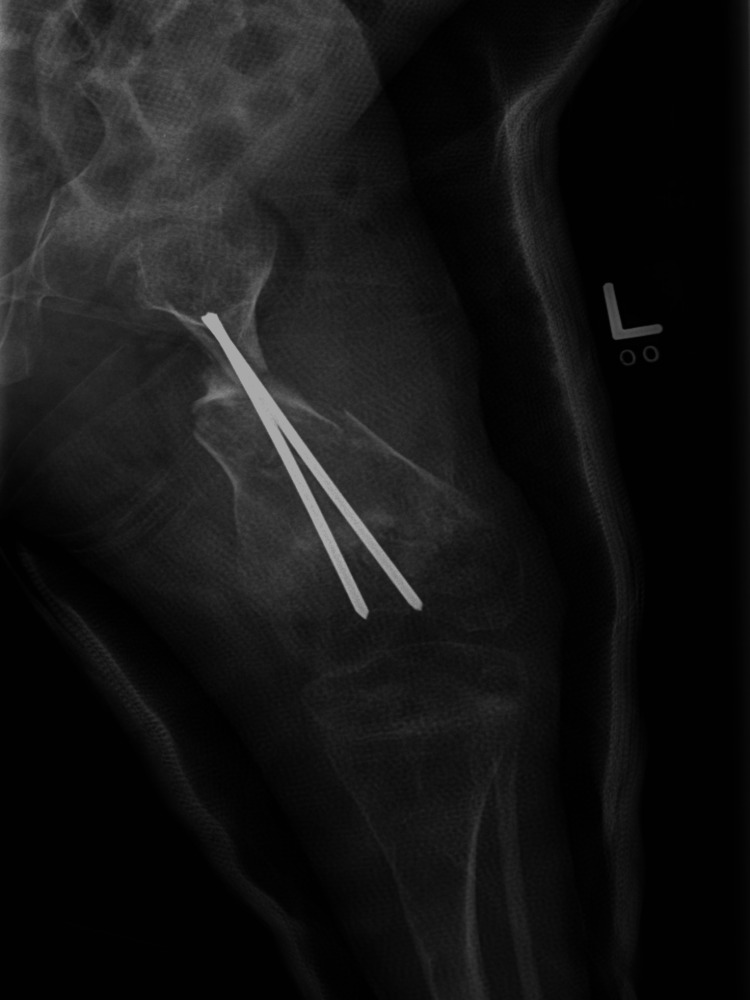
Immediate postoperative X-ray showed left femur osteotomy with rush nail fixation

The patient received his care postoperatively at the pediatric intensive care unit. Postoperative neurological examination revealed paralysis at T4-T7 levels, grade A of the American Spinal Injury Association (ASIA) impairment scale in which the impairment was complete. There wasn't any motor or sensory function below the level of injury. Three days postoperative X-ray images were inconclusive (Figure 2). Thus the decision was made to obtain spine CT four days after the operation; CT images were normal (Figure 3). Five days after the operation, MRI showed T4 high signal intensity within the spinal cord from level T3 to T7 due to ischemia signal changes in the dorsal spinal cord at T3-T7 (T4 apex of kyphosis) without evidence of compression at any level (Figure 4). There were no additional complications. Immediate postoperative X-ray showed left femur osteotomy with rush nail fixation (Figure 5). 

**Figure 6:**
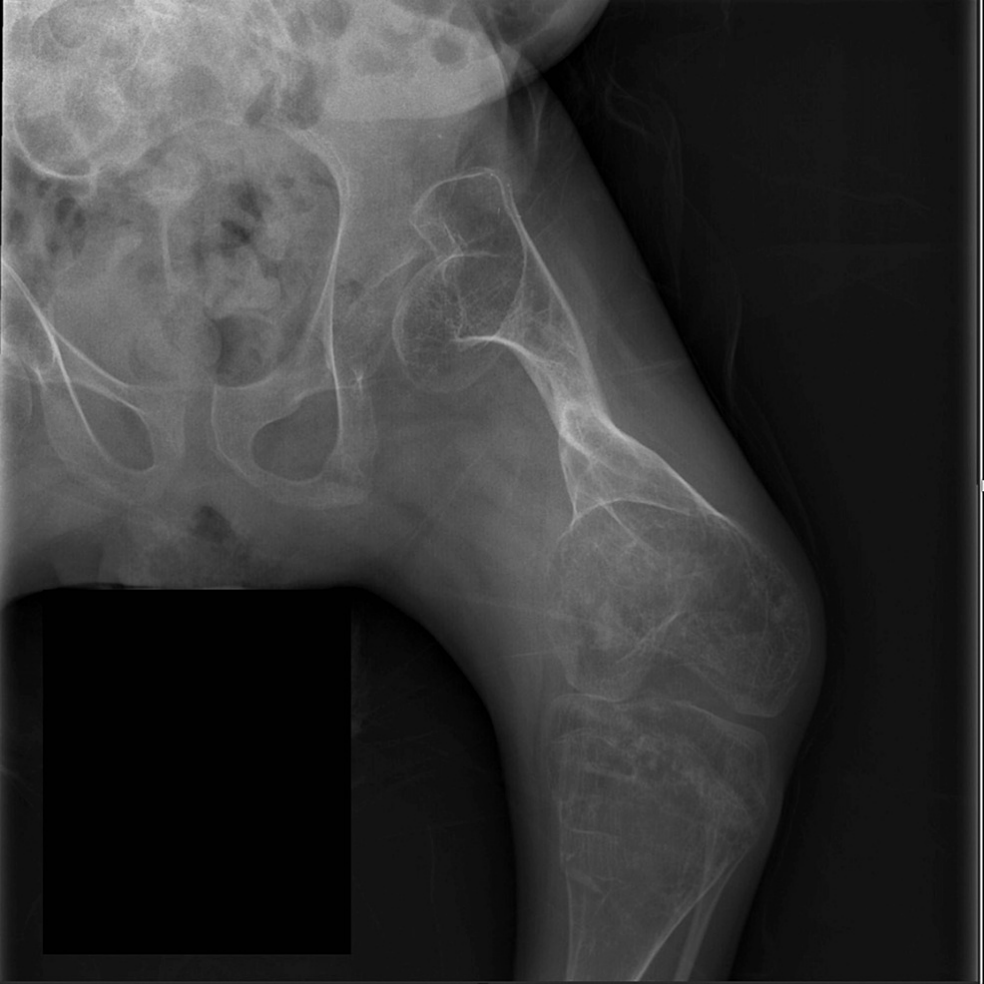
One year postoperative X-ray showed healed left femur osteotomy following rush nail removal

The patient went for rush nail removal six months later, and he was followed up with an X-ray one year postoperatively (Figure 6). The patient did very limited physical therapy due to osteoporosis. After follow-up, his neurological examination was improved to grade C ASIA, in which motor functions were preserved below the level of lesion, but more than half of the muscles had the power of grade 3. The time of recovery was 12 months. 

## Discussion

A key factor that may contribute to the development of postoperative spinal cord ischemia is blood loss during surgery [6], and a MAP of 60 mmHg or greater is usually required to guarantee enough blood flow to the essential organs. If the target MAP is not met, life-threatening consequences such as ischemia, stroke, and even sepsis may arise as a result. In such cases, boosting blood pressure above 85 mmHg with IV fluids and vasopressors is a life-saving procedure. Low MAP-induced ischemia might result in further damage to the previously injured spinal cord, resulting in a poor neurological outcome [6]. In our case, the lowest MAP measured was 45 mmHg, which may have contributed to the development of postoperative ischemia. 

Another aspect that could play a role is the use of general anesthesia for an extended duration of time. Although it is extremely rare, neurological complications, such as spinal cord ischemia, might occur as a result of epidural or general anesthesia. While the exact pathophysiology is not fully understood, intraoperative hypotension plays a fundamental role [7]. Nevertheless, no clear association was found in our case. Furthermore, while data reveals that anesthesia-induced spinal cord ischemia does not usually develop immediately after operation [7], positioning during spinal surgery is well known to cause postoperative neurological complications, and spinal cord infarction is one of them. The prone position is commonly associated with intraoperative hypotension and reduction in cardiac output [8]. However, our patient underwent a lower extremity correction procedure in the supine position. The prolonged supine position in kyphotic patients could result in physiological changes and soft tissue injury, including neuropathies in pressure areas where a reduction in perfusion leads to tissue ischemia and subsequent tissue breakdown. Prevention of injury in the supine position begins with proper positioning, reduced surgery time, and constant surveillance of the patient [9]. We believe that the position during the surgery played a role in our case. Finally, hypertension, diabetes, and obesity, along with a history of cerebral infarction and atherosclerotic lesions, have all been linked to spinal cord infarction in several studies; importantly, none of these have been exhibited by our patient [10].

## Conclusions

Multiple risk factors may contribute to the development of such a catastrophic event of spinal cord ischemia. Surgeons, physicians, and anesthesiologists need to be more aware of this complication, as it can lead to permanent patient disability. In addition, mean artery pressure should be closely monitored during the operation in order to prevent hypotension, and a close follow-up after the operation is of extreme importance in such instances.
